# Risk stratification to improve Pediatric Early Warning Systems: it is all about the context

**DOI:** 10.1007/s00431-019-03446-0

**Published:** 2019-09-04

**Authors:** Lara Teheux, Carin W. Verlaat, Joris Lemson, Jos M. T. Draaisma, Joris Fuijkschot

**Affiliations:** 1 Radboud Institute for Health Sciences, Amalia Children’s Hospital, Department of Pediatrics, Radboud University Medical Center, PO Box 9101, 6500 HB Nijmegen, The Netherlands; 2 Radboud Institute for Health Sciences, Department of Intensive Care Medicine, Radboud University Medical Center, Nijmegen, The Netherlands

**Keywords:** Early warning system (EWS), Trigger tool, Risk stratification, Health care quality, Patient safety

## Abstract

Early recognition of critically ill patients is of paramount importance to reduce pediatric mortality and morbidity. We created a risk stratification system combining vital parameters and predefined risk factors aimed at reducing the risk of unrecognized clinical deterioration compared with conventional Pediatric Early Warning Systems (PEWS). This single-center retrospective case cohort study included infants (gestational age ≥ 37 weeks) to adolescents (aged <18 years) with unplanned pediatric intensive care unit (PICU) admission between April 01, 2014, and February 28, 2018. The sensitivity in the 24 h prior to endpoint of the Pediatric Risk Evaluation and Stratification System (PRESS) was compared with that of the conventional PEWS and calculated as the proportion of study patients who received a high-risk score. Seventy-four PICU admissions were included. PRESS and PEWS sensitivities at 2 h prior to endpoint were 0.70 (95%CI 0.59 to 0.80) and 0.30 (95%CI 0.20 to 0.42) respectively (*p* < 0.001). Excluding patients with seizures, PRESS sensitivity increased to 0.75 (95%CI 0.64 to 0.85). Forty-nine patients (66%) scored positive on at least one high-risk factor, and “worried sign” was scored in 31 patients (42%).

*Conclusion*: Risk stratification seems advantageous for a faster detection of clinical deterioration, providing opportunity for earlier intervention.

**Table:** 

**What is Known:** *• Prompt detection of clinical deterioration is of essential importance to reduce morbidity and mortality.* *• Conventional Pediatric Early Warning Systems (PEWS) have limited sensitivity and a short window of detection of 1 to 2 h.*	
**What is New:** *• Risk stratification based on context factors allows earlier identification of patients at risk, well before deviation of vital signs.* *• Risk stratification combined with continuous monitoring of deteriorating trends in vital signs could lead to the development of next-generation warning systems achieving true patient safety.*

**Electronic supplementary material:**

The online version of this article (10.1007/s00431-019-03446-0) contains supplementary material, which is available to authorized users.

## Introduction

Pediatric Early Warning Systems (PEWS) are used in most modern hospitals around the world, aimed at improving recognition of clinical deterioration in order to reduce mortality and morbidity. PEWS generally consist of a number of vital signs and other clinical indicators suggesting critical illness, often accompanied by an algorithm with rules for escalation and de-escalation of care.

Despite the considerable amount of literature concerning PEWS, the evidence supporting their effect is limited and various systematic reviews failed to demonstrate more than a positive trend on clinical outcomes [1, 4, 5, 15, 24]. A recent prospective multicenter randomized controlled trial of PEWS found no decrease in all-cause hospital mortality, although a composite outcome measure showed a significant reduction of late intensive care admissions [17]. A particular concern is the lack of standardization in PEWS, imposing a major challenge in comparing systems and limiting generalizability of study results. Studies from the UK and the Netherlands have identified largely different PEWS in pediatric units, with a considerable number remaining self-designed and unvalidated [10, 20, 26]. Variations in reference range of vital signs in pediatric patients provide an additional challenge. In contrast to these challenges, qualitative studies demonstrate positive effects of PEWS on situational awareness and overcoming communication obstacles such as hierarchical barriers [2, 3].

We anticipated that the rather limited sensitivity of PEWS could be a result of the narrow focus on vital signs, not addressing other factors that may also predict clinical deterioration. Therefore, in 2014, we designed and implemented the Pediatric Risk Evaluation and Stratification System (PRESS) [12], which integrates (1) a selection of predefined risk factors with (2) a previously validated PEWS [13] and (3) patient’s responsiveness (AVPU score). Based on a combination of these components, patients are stratified into three risk categories: high, medium, and low risk. High-risk patients (so called watchers) receive proactive checks upon their clinical condition. A recent qualitative study illustrated that the PRESS enhanced situational awareness in physicians and nurses [7], but to date the effect of the PRESS on timely recognition of clinical deterioration has not yet been quantified.

The primary goal of this study was to quantify the value of risk stratification by the PRESS in predicting clinical deterioration in hospitalized pediatric patients. The secondary aims were to provide new insight into the value of individual risk factors, to explore the impact of risk stratification on clinical outcomes, and to investigate factors in protocol adherence.

## Methods

### Setting

The Radboudumc Amalia Children’s Hospital is a Dutch tertiary referral hospital with three pediatric wards (72 beds) where specialized care is offered by a broad spectrum of surgical and non-surgical pediatric specialists and nurses. Monitoring of vital parameters and various emergency medical interventions (e.g., fluid challenge and supplemental oxygen) can be performed in the ward. Critically ill children can be transferred to a 10-bed pediatric intensive care unit (PICU). PICU mortality rate between 2014 and 2018 was 2.2%.

**Fig. 1:**
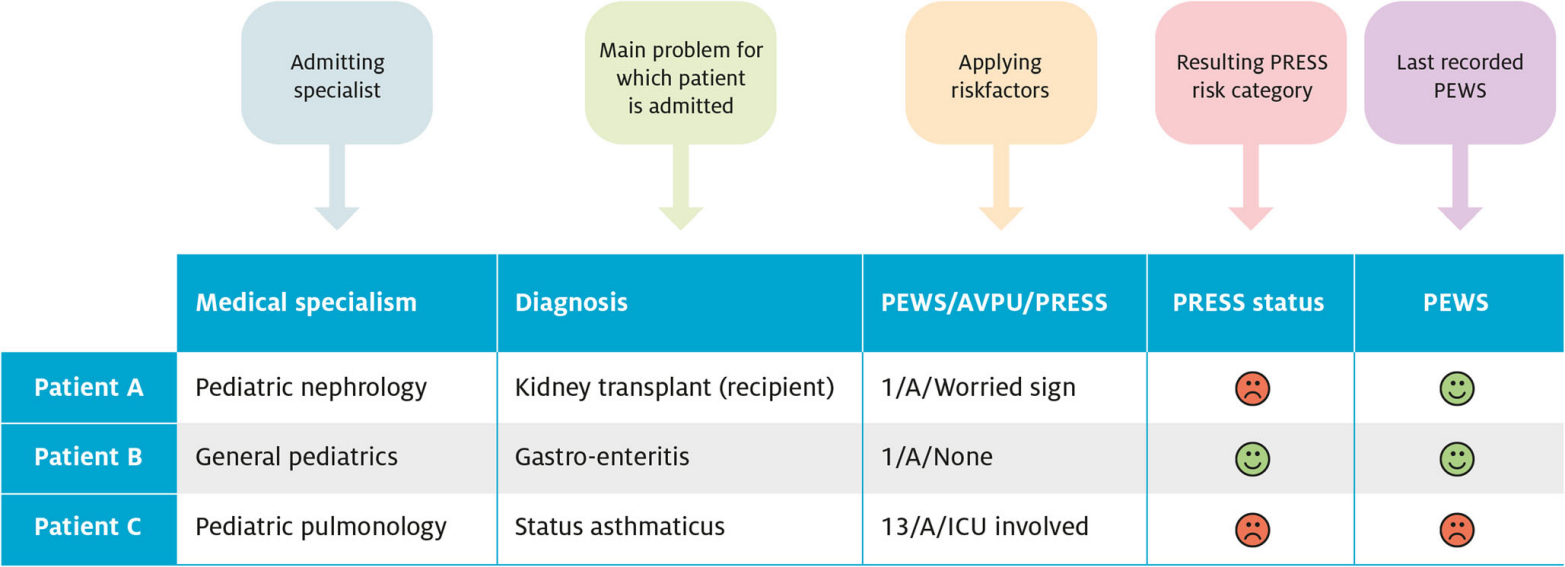
Visualization of PRESS in the electronic health record system. A real-time dashboard of the ward shows the PRESS status and last recorded PEWS by a colored smiley (red indicating high-risk PRESS or PEWS score above the threshold, orange indicating medium-risk PRESS or PEWS score elevated but not above the threshold, green indicating standard-risk PRESS or normal PEWS) and is fully integrated in the hospital’s electronic health record system (Epic, Systems Corp, Verona, WI). PRESS is updated manually at least once a day, while the most recent PEWS is automatically uploaded to the dashboard allowing a change in the PEWS score to be detected irrespective of the PRESS score. The dashboard displays how the PRESS risk category was determined based on the three components (1) PEWS, (2) AVPU, and (3) predefined PRESS risk factors, allowing professionals to interpret the different components easily. PEWS, Pediatric Early Warning Score; AVPU, alert-verbal-responsive to pain-unresponsive; PRESS, Pediatric Risk Evaluation and Stratification System; ICU, intensive care unit

All patients in the ward are routinely scored by the nursing staff using our local PEWS [13] (a modified version of the Bedside PEWS [16]) and AVPU three times a day or more frequently in case of elevated scores and at least once daily when vital signs are stable for at least 24 h. The predefined risk factors are scored by the attending physician at least once daily during rounds. The predefined high-risk factors scored by PRESS include “worried sign” (physician, nurse, or family concern), “ICU involvement” (consultation of ICU specialists), “high-risk treatment” (complex and/or potentially risky procedures), “transferred patients from general hospitals” (referrals or transfers from general hospitals during on-call hours), and “abnormal pH or lactate” (≤ 7.20 and ≥ 4.0 mmol/l respectively). Patients are scored high risk if PEWS is ≥ 8 [13], if an AVPU score is “P” (responsive only to pain) or “U” (unresponsive), and/or if one high-risk factor is considered positive. The PEWS score and PRESS risk category are simplified into color codes. This results in a live dashboard, which assists the attending professionals to quickly identify watchers in the ward (Fig. 1). Watchers receive more proactive checks from professionals according to previously published standard operating procedures [7]. A validity indicator (indicating if a PRESS risk stratification has been performed that day) was added to the dashboard in 2017, to facilitate the detection of invalid PRESS scores and to enhance protocol adherence.

### Study design

We conducted a single-center retrospective case cohort study. The performance of the PRESS was studied retrospectively over the period from April 1, 2014, to February 28, 2018. We included patients between 37 weeks gestational age and 18 years who, at any time during their admission to the pediatric ward, had deteriorated towards the endpoints “unplanned PICU admission” or “cardiopulmonary arrest.” Patients with a “do not resuscitate order” were excluded. Additionally, PICU admissions of a non-urgent nature were excluded, as well as readmissions to the PICU within 24 h (since these patients would always be marked as watcher patients according to standard operating procedures). Patients who were admitted to the pediatric wards for less than 2 h prior to reaching the defined endpoint were excluded, as this time period was considered insufficient for early detection. Exclusion was confirmed after review by a panel of two researchers (LT/JF).

The patients in this study were recruited from a list of unplanned PICU admissions drawn from the hospital’s electronic health record system. For specificity analyses, a random control sample in a 1:1 ratio to the study admissions was drawn from the list of pediatric ward admissions in 2017. The procedures of this study were in accordance with local ethical committee guidelines.

### Data analysis

The PEWS scores and PRESS risk stratification in the 24 h prior to the endpoint were studied retrospectively based on data extracted from the electronic patient files. PRESS and PEWS scores were considered expired after 24 and 8 h respectively, in accordance with local protocol. Patients with a valid PRESS and PEWS score at the endpoint were included in the performance analyses. The sensitivity was calculated as the proportion of patients with cardiopulmonary arrest or unplanned PICU admission that received a high-risk PRESS stratification or PEWS above threshold (≥ 8). The specificity was determined as the proportion of control admissions in the general ward that did not receive any high-risk stratification during the total admission duration.

To assess the value of separate risk factors, we analyzed which risk factors were most commonly scored. Clinical outcomes in watcher and non-watcher patients were compared using the Pediatric Risk of Mortality III score (PRISM III) [18] and Pediatric Index of Mortality 2 score (PIM2) [23] mortality rate, resuscitation, and PICU interventions in the first 24 h after PICU admission. Additionally, protocol adherence was assessed with a comparison of the daily scoring of the PRESS between cohort pre- and post-implementation of the validity indicator.

Data analyses were performed using SPSS software (version 22.0). Summary statistics were obtained using the *t* test comparison of means, the Mann-Whitney *U* test, Pearson chi-square test, or Fisher’s exact test, as appropriate. A 2-sided *p* value of < 0.05 was considered significant.

## Results

### Patient characteristics

**Fig. 2:**
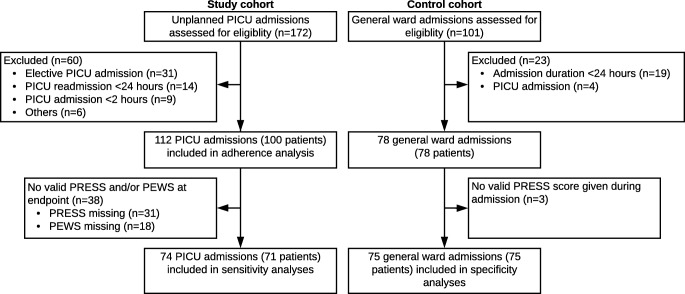
Flow diagram of inclusion. PEWS, Pediatric Early Warning Score; PRESS, Pediatric Risk Evaluation and Stratification System; PICU, pediatric intensive care unit

One hundred seventy-two unplanned PICU admissions were assessed for eligibility of which 98 were excluded (Fig. 2). Seventy-four admissions were included for sensitivity analysis. No additional patients were identified based on the endpoint cardiopulmonary arrest in the general ward. In order to include 75 controls, a total of 101 general ward admissions were assessed for eligibility (Fig. 2). The study cohort contained significantly less surgical patients. Further patient characteristics are described in Table 1.

**Table 1:** Patient characteristics

Patient characteristics	Cohort		
	Study (*n* = 74)	Control (*n* = 75)	*p*
Gender, no. (%)			NS
Male	38 (51)	43 (57)	
Female	36 (49)	32 (43)	
Age, Mdn (IQR)	2.0 (0.2–11.4)	1.9 (0.4–10.9)	NS
Age group, no. (%)			NS
0–3 months	19 (26)	15 (20)	
≥ 3–12 months	12 (16)	15 (20)	
≥ 1–4 years	15 (20)	15 (20)	
≥ 4–12 years	10 (14)	15 (20)	
≥ 12 years	18 (24)	15 (20)	
Discipline, no. (%)			0.009
Surgical	12 (16)	27 (36)	
Non-surgical	62 (84)	48 (64)	
Admission duration (days), Mdn (IQR)	2 (1–6)	4 (2–9)	NS
Severity of illness			
PIM2 mortality risk, Mdn % (IQR)	2.1% (1.3–5.1)	n/a	
PRISM III, Mdn (IQR)	10 (7–14)	n/a	
Mortality, no. (%)	5 (7)	0 (0)	

*IQR* interquartile range, *Mdn* median, *n/a* not applicable, *NS* nonsignificant, *PIM2* Pediatric Index of Mortality 2, *PRISM III*, Pediatric Risk of Mortality III

### Sensitivity and specificity

**Fig. 3:**
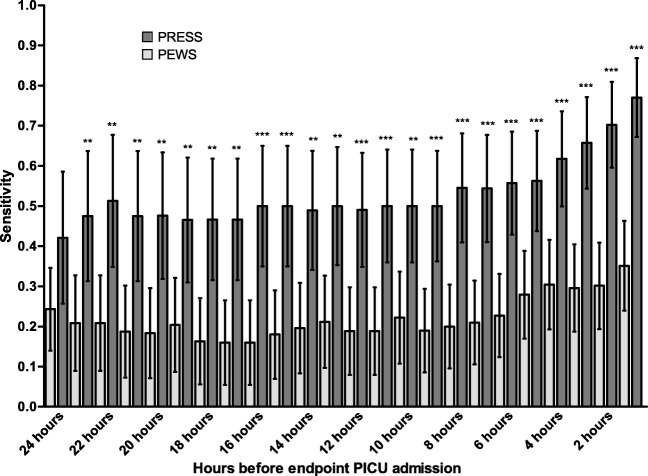
Progression of PRESS and PEWS sensitivity over time preceding the endpoint PICU admission. The graph represents the sensitivity of PRESS and PEWS (dark and light gray respectively) at each hourly time point prior to PICU admission. 95% confidence intervals are displayed with error bars. **Difference between PRESS and PEWS at a time point significant at the 0.01 probability level. ***Difference between PRESS and PEWS at a time point significant at the 0.001 probability level

PRESS sensitivity was significantly higher than PEWS sensitivity in the 23 h prior to endpoint PICU admission (Fig. 3). PRESS and PEWS sensitivities at 2 h prior to endpoint were 0.70 (95%CI 0.59 to 0.80) and 0.30 (95%CI 0.20 to 0.42) respectively (*p* < 0.001) and 0.62 (95%CI 0.49 to 0.73) and 0.30 (95%CI 0.20 to 0.43) at 4 h to endpoint (*p* < 0.001). No significant differences in PRESS sensitivity were found in the subgroup analyses based on gender, age, and admitting specialism. However, PRESS sensitivity was significantly lower in patients with convulsions compared with those with all other diagnoses (0.29 versus 0.75, *p* < 0.05, Online Resource Table Ia). The PRESS specificity was 0.67 (95%CI 0.55–0.77).

Forty-nine patients (66%) scored positive on at least one high-risk factor (Online Resource Table Ib) “Worried sign” was the most prominent factor (42%). One patient (1%) was flagged exclusively by an elevated PEWS score. One patient (1%) was stratified as high risk based on “abnormal pH or lactate,” while retrospective analysis of laboratory results revealed that 15 patients (20%) could have been identified based on this risk factor. Three patients (4%) were missed because the PRESS stratification was not changed manually after the lab results were known, and all other patients with abnormal lab results scored positive on another high-risk factor.

### Clinical outcomes

Overall PICU mortality in the study group was 7%, higher than expected according to PIM2 and PRISM III (Table 1). A non-significant difference in PICU mortality was found between watcher and non-watcher patients (4% vs 14% respectively, odds ratio 0.25 (95% confidence interval 0.04–1.64)).

### Protocol adherence

Protocol adherence increased significantly after implementation of the validity indicator. For more detailed results, see the Online Resource.

## Discussion

### Key findings

The results of this study show that the PRESS reaches sensitivity comparable with that of other validated PEWS [13, 16] considerably earlier (from 4 h preceding the endpoint) allowing faster detection and proactive checks to prevent further deterioration. The sensitivity of the PRESS increased over the hours leading up to the event.

This study demonstrates that context risk factors are highly potent in identifying patients at risk for clinical deterioration attributing to 96% of PRESS sensitivity, while PEWS sensitivity remained remarkably low. It is likely that risk stratification based on pre-existing factors allows for earlier identification of patients at risk, well before deviation of vital signs. In our study population, abnormal lactate and/or pH could identify 20% of patients at risk, consistent with various previous studies supporting the use of lactate as a predictor in pediatric patients with sepsis [6, 14, 22]. It should be noted that these results do not validate these laboratory tests as screening tools in the general population, but should be put in perspective as a tool to raise situational awareness in risk patients.

It should be highlighted that “worried sign” was the most preeminent risk factor, consistent with previous studies that noted adding nurses’ worry as a criterion raised sensitivity of EWS [8]. The intuitive knowing that something is wrong is often based on unconscious observations and plays an important role in clinical decision-making [19]. Previous studies support the assumption that health professionals’ worry often precedes the deviation of vital signs [9, 21, 25]. Our data justifies the use of worried sign as an independent factor to escalate levels of care.

Surprisingly, the PEWS was found to have a substantially lower sensitivity than reported in earlier validation studies of the Bedside PEWS [16] and our modified PEWS [13]. We stipulated that this inconsistency would be due to our “work as done” analyses in which we included only actual calculated PEWS scores (but could be given up to 8 h previously), in contrast to the other validation studies that use reconstructed and pooled scores. A post hoc analysis including scores given at maximum 2 h prior to endpoint (*n* = 29) did however not lead to significant improvement of PEWS sensitivity. Another possible explanation for the underexpectation performance of PEWS is that earlier detection by the PRESS has influenced clinical decision-making, leading to earlier transfer to the PICU prior to the rather short window of detection (one to maximally 2 h) of the PEWS. In general, PEWS sensitivity is also limited by the rather broad intervals of age-specific reference values. Patients can shift from the p10 range to the p90 range of normal vitals without leading to an alarming PEWS score, and hence, trends in deterioration can easily be missed.

A remarkable result was that the mortality was higher than expected based on PIM2 and PRISM III prediction models and higher compared with the general local mortality rate. It seems likely that the unplanned nature of the PICU admissions, a known risk factor, contributed to this high mortality. Additionally, a post hoc analysis revealed that 82% of study patients and 100% of patients with fatal outcome were suffering from a complex chronic condition, another well-established risk factor for mortality [11, 27]. Clinical outcome measures did not differ significantly between watcher and non-watcher patients, suggesting equality of care and opposing neglect of non-watchers. As one would expect worse clinical outcome measures among the watchers, this could point to positive effects upon outcomes of risk stratification and proactive and intensified follow-up of these patients. However, our sample size was inadequate to assess infrequent outcome measures such as mortality; therefore, these results should be interpreted with caution.

### Limitations

Three main limitations to this study can be identified. First, the interpretation of these results is limited as we performed a single-center study in a tertiary center using a unique stratification system. A considerable amount of patients were suffering from a complex chronic disease, limiting the external validity of our results. However, the contribution of risk factors to sensitivity of the PRESS is strong and it seems unrelated to the local context. Lessons learned from this study can be applied to PEWS in other contexts and are not solely restricted to pediatric practice.

Second, a considerable amount of data was missing from the patient files due to suboptimal protocol adherence. To reduce the effect of missing data, we excluded all patients with expired PRESS or PEWS scores. However, this provides a potential bias if severity of illness would influence the risk of missing data. Prospectively obtained data could have provided more complete data and therefore would have limited the possible influence of differences in patterns of missing data in patients with high and low PRESS and PEWS scores.

Final, the “work as done” analysis, in which scores were considered valid for several hours in accordance with local protocol, likely resulted in an underestimation of the theoretical PEWS and PRESS sensitivity, as it is well established that the sensitivity of PEWS decreases fast each extra hour preceding endpoint. Early warning is highly dependent on detecting deviation in trends of vitals, but in normal clinical practice, measurements are done infrequently and highly dependent on the time invested by nursing staff to complete measurements resulting in underperformance of the systems compared with theoretical capabilities.

### Implications

The results of this study indicate that risk stratification can be of benefit to the early detection of patients at risk of clinical deterioration. In current practice, risk factors such as worried sign are often integrated in the PEWS as one of the scoring items, only leading to escalation of care when other items start to deviate and neglecting the significance of context factors as a separate predictor. Our study legitimizes a shift of focus to systems with risk stratification based on multiple context factors, each of which has different powers to (directly) escalate levels of care.

Continuous registration of vitals (e.g., by biomedical sensors) and detection of trends in personalized vital patterns (e.g., by intelligent software that analyzes and personalizes resulting data flow continuously) may increase EWS performance in clinical practice. Together with risk stratification and proactive follow-up of all watchers (including the ones with normal vitals) as done in the PRESS, this could lead to the development of a next-generation warning system that truly contributes to patient safety [28, 29].

## Conclusions

This study has shown that early warning systems’ performance can be enhanced with risk stratification. These findings warrant a shift of focus to systems that combine context factors with vital signs in order to achieve earlier detection and intervention in patients at risk.

## Electronic supplementary material

ESM 1 (PDF 253 kb)

## Abbreviations

AVPU: Alert, verbal, responsive to pain, unresponsive
PEWS: Pediatric Early Warning System
PICU: Pediatric intensive care unit
PIM2: Pediatric Index of Mortality 2
PRESS: Pediatric Risk Evaluation and Stratification System
PRISM III: Pediatric Risk of Mortality score III
