# Supplementary material for: Risk stratification to improve Pediatric Early Warning Systems: it is all about the context
Source: Eur J Pediatr. 2019 Sep 4;178(10):1589–96. doi: 10.1007/s00431-019-03446-0 (PMC6733815; doi:10.1007/s00431-019-03446-0)
Supplement: Supplementary file 1 — (PDF 253 kb) [file 431_2019_3446_MOESM1_ESM.pdf]

SUPPLEMENT

Table Ia Results: subgroup analysis

**Patient subgroup**		**Sensitivity†**			
		**PRESS**		**PEWS**	
	No		p		p
Gender.			NS		NS
Male.	38	0.72		0.34	
Female.	36	0.68		0.26	
Age group.			NS		NS
0-3 months.	19	0.68		0.37	
≥3-12 months.	12	0.67		0.09	
≥1-4 years.	15	0.73		0.13	
≥4-12 years.	10	0.70		0.60	
≥12 years.	18	0.72		0.33	
Discipline.			NS		NS
Surgical.	12	0.67		0.18	
Non-surgical.	62	0.71		0.32	
Diagnosis.			NS		NS
Airway.	6	1.00		0.00	
Breathing.	34	0.79		0.41	
Circulation.	14	0.64		0.29	
Disability.	16	0.50		0.19	
Other.	4	0.50		0.25	
Acute convulsions			0.02		NS
Convulsions	7	0.29		0.14	
No convulsions	70	0.75		0.32	

† Sensitivity at two hours prior to the endpoint PICU admission.
Abbreviations: PRESS, Pediatric Risk Evaluation and Stratification System; PEWS, Pediatric Early Warning Score; NS, non-significant

Table Ib Results: PRESS factors

PRESS factor	All patients (n=74)		High risk patients† (n=51)	
	No	%	No	%
PRESS.	74	100	-	-
High risk.	51	69	-	-
PEWS above threshold (≥ 8).	13	18	13	26
AVPU score.				
Unresponsive.	0	0	0	0
Pain.	2	3	2	4
High risk factors.	49	66	49	96
Worried sign.	31	42	31	61
ICU involvement.	13	18	13	26
High-risk treatment.	1	1	1	2
Transferred patient.	3	4	3	6
pH or lactate.	1	1	1	2
Laboratory tests‡.	58	78	42	82
pH ≤7.20.	5	7	3	6
Lactate ≥4.0 mmol/l.	13	18	11	22
pH and lactate abnormal.	3	4	2	4

Description of the factors scored at the time of the last validated PRESS score before the endpoint PICU admission. The median time to endpoint was five hours [IQR 2–16].
† Number and proportion of patients in the high risk group with the concerning PRESS factor.
‡ Number and proportion of patients in which laboratory test of pH and lactate were performed and were abnormal respectively. The PRESS score needs to be manually updated to include these results before a high risk stratification was noted, which was done in one patient. In three patients the score was not updated. All other patients with abnormal lab results scored positive on another risk factor.
Abbreviations: PRESS, Pediatric Risk Evaluation and Stratification System; PEWS, Pediatric Early Warning Score; AVPU, Alert – Verbal – Responsive to Pain – Unresponsive; ICU, Intensive Care Unit.

Table Ic Results: Clinical outcomes

Outcome indicators	PRESS risk category†			Odds ratio‡ (95% CI)
	**High** (n=52)	**Medium/standard** (n=22)		
Mortality. No (%)	2 (4)	3 (14)	NS	0.25 (0.04–1.64)
Severity of disease scores. Mdn [IQR]				
PIM2 mortality risk.	2.1% [1.3–5.5]	2.1% [1.2–4.9]	NS	
PRISM III.	10 [7–13]	10 [8–15]	NS	
Resuscitation§. No (%)			NS	0.67 (0.15–3.10)
Ventilation and compressions.	2 (4)	2 (9)		
Ventilation support only.	3 (6)	1 (5)		
PICU support¶. No (%)				
Intubation.	16 (31)	6 (27)	NS	1.19 (0.39–3.59)
Respiratory support.			NS	2.06 (0.74–5.69)
Invasive ventilation.	16 (31)	6 (27)		
Non-invasive ventilation	2 (4)	0 (0)		
High flow oxygen.	17 (33)	5 (23)		
Circulatory support.			NS	1.98 (0.69–5.67)
Vasoactive medication and fluids.	11 (21)	3 (14)		
Fluid resuscitation	14 (27)	4 (18)		

† PRESS risk category at two hours prior to the endpoint PICU admission.
‡ Odds ratio for outcome indicator in high risk category versus medium or standard risk category.
§ Resuscitation necessary in the general pediatric ward or within 24 hours post PICU admission. All ventilation support was given with mask and balloon in the general pediatric ward. Two patients required ventilation support and thorax compressions in the general pediatric ward and two patients in the PICU.
¶ Within 24 hours post PICU admission.
Abbreviations: NS, nonsignificant; PIM2, Paediatric Index of Mortality 2; PRISM III, Pediatric Risk of Mortality III; No, number; Mdn, median; IQR, interquartile range.

Table Id Results: Adherence pre- and post-implementation of the validity indicator

Adherence indicators	Cohort		
	Pre-implementation	Post-implementation	
PRESS protocol adherence	(n=79)	(n=32)	
Daily scoring. No (%)	28 (35.4)	18 (56.3)	0.045
Proportion of days scored. (95% CI)	0.59	0.83	0.002
Sensitivity†.	(n=51)	(n=32)	
PRESS (95% CI)	0.75 (0.63-0.86)	0.61 (0.41-0.81)	NS
PEWS (95% CI)	0.38 (0.25-0.51)	0.13 (0.00-0.27)	NS

† Sensitivity of PRESS and PEWS at 2 hours prior to endpoint PICU admission.
Abbreviations: PRESS, Pediatric Risk Evaluation and Stratification System; NS, nonsignificant; PEWS, Pediatric Early Warning Score.
